# Gleanings from Camp and Hospital

**Published:** 1874-05-01

**Authors:** F. K. Bailey

**Affiliations:** Knoxville, Tenn.


### MEDICAL EXAMINER.

A

*Semi-Monthly Journal of Medical Sciences.*

EDITED BY N. S. DAVIS, M.D., AND F. H. DAVIS, M.D.

No. IX. CHICAGO, MAY 1, 1874. VOL. XV.

### Original Communications.

### GLEANINGS FROM CAMP AND HOSPITAL.—II.

BY F. K. BAILEY, M.D., KNOXVILLE, TENN.

UPON the formation of camps, and the aggregation of hundreds and thousands of men from different localities and the varied walks in home-life, in our place, with none of the appliances of comfort enjoyed at home, and, superadded, the thousand and one conditions incident only to the army in the field, it was to be expected that disease would assume different forms, according to circumstances.

May 11th, 1861, about one thousand men reported at Joliet, in Illinois, for the purpose of organization into a regiment of soldiers. All ages, from the beardless boy of sixteen to the matured man of well nigh to sixty, composed this collection. After about ten days, it was found that twenty or more were laboring under diphtheria. On investigation, it was found that the victims were men from localities where the disease had been prevalent during the winter and spring. No cases occurred among those who came from places free from it. This afforded an interesting illustration of the co-operation of *predisposing* and *exciting* causes in the production of disease. (*Williams' Principles*—" Etiology.")

Another striking instance, of a like character, occurred at Cape Girardeau, Mo. The 20th Ill. Infantry arrived at that place July 10th, and encamped at once upon the bank of the Mississippi. The spot chosen was rather flat and basin-like. It was dry, and, in a land where no rain falls the location would have been faultless. But, alas! it was otherwise. In a few days there was a copious shower, and many of the tents "swam in water." With this admonition, no hint was taken to move. Soon the sick lists began to enlarge, and strong men were prostrated by fever.

The first death in the regiment occurred before the first of August. Still, comparatively few were sick.

Having been accustomed to malarial influence at their homes in the Prairie State, they were not obnoxious to ordinary local causes; besides, the daily routine of duty only included the usual humdrum of lazy camp-life. But, during the first week in August, an alarm was given, and the enemy reported as advancing on the place. Orders came, shortly after taps, to prepare for a move. A greater portion of the night was consumed in the hurry and bustle incident to packing up for a supposed long march. Early morning found us upon an elevation overlooking the river, and hardly a stone's throw from the old camp. To fortify was the command; and in a few hours nearly two thousand men were at work with pick and shovel, digging deep trenches, the earth from which was piled into high embankments. The excited men, incited by the more excited officers, thus worked two nights and a day. A line of defence was thrown up in an incredibly short period of time. Intelligence was received that it was only a scare.* Excitement was succeeded by depression, from fatigue and want of sleep, and at once there were hundreds attacked with chills. The exposure, in a low camp, to vicissitudes of heat and wet, served as a predisposing cause, to be converted into the disease itself by the same physical exertion as above stated. From this time, fever of a low grade became common, and many sunk to a soldier's grave.

There is much reason to believe that the exposure to the air of an extensive surface of earth is a cause of disease in any locality and under any circumstances. The exposure of fresh earth by the plow, on our vast prairies, is considered as productive of miasmatic disease, by reason of the decay of grass roots. The grading of railroad tracks, and excavations for laying foundations for buildings, are cases in point.

In the summer of 1870, a large store was built in this city, and over 13,000 square feet of surface was exposed, to the depth of eight or ten feet, in order to secure a solid foundation. The gentleman who supervised the work was, although large and stout-appearing, a sufferer from a chronic torpidity of the portal system. He was exposed a greater part of every day to any emanation which might have arisen, and under a hot summer sun. He at length was taken with severe pain in the head; a loss of power to calculate in his building plans; and from some real or fancied error being found in the details of the work, he became so chagrined that he shot himself through the head.

The spot referred to had for some years been an open area, where horses had been tied by country people, and a great amount of impurity had become incorporated with the soil. It is not improbable that laborers, merely digging upon the same ground, escaped because of the exercise; whereas a mere superior only stood

* I am told by a gentleman in this city who was connected with Gen. Pillow's forces, that the nearest that they went to Cape Girardeau was sixteen miles.

about, without any exertion sufficient to cause sweating. A common laborer will endure exposure and fatigue, from physical exertion alone, better than the man who does no work, but has the mental strain of responsibility to bear.

Last summer an addition was made to the Deaf and Dumb Asylum in this city, and upon ground where the kitchen and laundry slops had been thrown for many years. An excavation was dug to the depth of several feet for a basement story, and a great quantity of earth removed and spread out upon one corner of the grounds not far distant. Before the building was finished quite a number of the inmates sickened with a low grade of fever, while no other cases occurred in the city.

There is no doubt but that quite a proportion of fever cases occurring during the late war resulted from undue exposure to causes as above indicated.

Typho-malarial fever appears to be an intensifying of paludal influences, coupled with other causes of a physical and mental nature. Exposure in cold weather was a fruitful cause of the camp dysentery which became so prevalent in our army immediately after the capture of Fort Donelson.

During the approach upon that famous stronghold no fires were allowed; and the whole army was exposed for days to a continuous storm of rain, which froze as it fell, and consequently every man was more or less wet. Dysentery and diarrhœa at once seized upon those who had escaped the bullet; and after the forces had retired to their respective camps their tents were found tattered and torn by bullets and hard usage. The rains of the last half of February were frequent, and exposure was extreme.

During the week consumed in sailing up the Tennessee River to Pittsburgh Landing and Savannah, there was great suffering, and but few were in good health. In consequence of a long-continued exposure of the cutaneous surface to cold and dampness, the hepatic system was loaded, and the intestinal mucous membranes became diseased.

For five or six months after the Fort Donelson exposure, I was not free, for many days at a time, from a hæmorrhagic discharge from the bowels. This condition originated from exposure to cold and dampness during the winter of 1861–2, while our command was at Bird's Point, Mo., together with our exertion in closing up the regimental hospital, after the army left for Fort Henry. I did not arrive at Fort Donelson till February 16th, the day of the surrender. From this time till March 4th, it was necessary to visit the camp daily, and prescribe for a great number who were complaining. A regimental hospital was opened in the village of Dover, where we had a goodly number of my sick patients.

Surgeon Goodbrake, of the 20th, was with the command through the Fort Henry and Fort Donelson campaigns; and, if I remember correctly, his health suffered very materially, in consequence of exposure and the extraordinary physical exertion, which were absolutely unavoidable in those memorable days and nights. From March 13th till the 6th of April following, the forces were somewhat benefited by such a respite from severe duty as could be obtained in cool and rainy weather, with poor tents, pitched in the mud.

But the incidents of camp life, which are yet so fresh in memory, and associated with our sojourn at Savannah before and after the battle of Shiloh, will afford material for another article in this series.

