# Correction

**Published:** 1874-05-15

**Authors:** 


CORRECTION.—In the article by F. K. Bailey, in May 1st No. EXAMINER, page 211, fourteenth line from bottom of second column, instead of *my* read *very*.

